# Exploring the taste code of light-withered tea: the mechanism of nonvolatile metabolite changes in LED light-withered oolong tea (*Camellia sinensis*)

**DOI:** 10.1016/j.fochx.2025.102873

**Published:** 2025-09-10

**Authors:** Qiuming Li, Jihang He, Xinru Yu, Yongpeng Wu, Kang Li, Zongjie Wu, Yucheng Zheng, Yun Sun, Hongzheng Lin

**Affiliations:** a Key Laboratory of Tea Science, College of Horticulture, Fujian Agriculture and Forestry University, Fuzhou 350002, China; b Fujian Vocational College of Agriculture, Fuzhou, Fujian 350303, China; c College of Tea and Food Sciences, Wuyi University, Tea Engineering Research Center of Fujian Higher Education, Tea Science Research Institute of Wuyi University, Wuyishan 354300, China; d Wuyishan Yongpeng Tea Industry Co., Ltd, Wuyishan 354300, China

**Keywords:** Oolong tea, LED combination light, Withering, Taste

## Abstract

Rainy weather hinders normal sunlight withering of oolong tea, which prevents the development of its high-quality characteristics. This study used representative oolong tea varieties (“Shuixian,” “Ruixiang,” and “Chunlan”) and applied multi-wavelength LED combination light, with control group for sensory and metabolomics comparison experiments. Sensory omics results showed that LED light-withered oolong tea has a more mellow taste, with deeper orange-yellow and orange-red hues. Widely targeted metabolomics analysis revealed that LED light withering affected the taste of oolong tea by promoting transformation of key bitter-related metabolites, including flavonoids (Kaempferol-3-O-rutinoside, Epigallocatechin, Kaempferol, Robinin), alkaloids, bitter amino acids, and oligopeptides. These changes were accompanied by varying levels of umami and sweetness, resulting in more mellow taste. This research clarifies the relationship between metabolite changes and flavour enhancement following LED light withering, providing systematic theoretical foundation for the controlled light-withering of oolong tea under rainy conditions and its quality improvement.

## Highlights

• LED light withering process significantly improves oolong tea's flavour profile.
• LED light withering enhances soluble sugars and alkaloid levels in oolong tea.
• Flavonoid conversion drives mellow flavour in LED light withered oolong tea.
• LED lighting enhances oolong tea withering quality in rainy conditions.

## Graphical abstract

**Figure:**
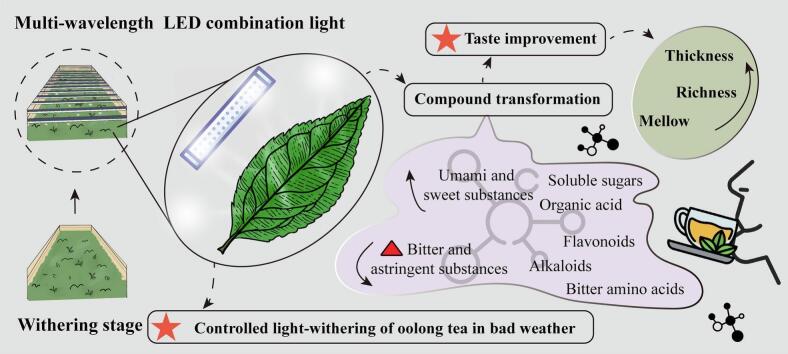

## 1 Introduction

*Camellia sinensis* is the perennial evergreen plant of the *Theaceae* family, whose leaves and buds are exclusively used to produce teas (e.g., oolong). Oolong tea is produced primarily in Fujian Province, China, and is known for its rich, sweet, and mellow flavour profile (Li et al., 2023). The unique flavour of oolong tea is closely linked to both its processing technique and the quality of the fresh leaves. Each year, the main production phase of oolong tea takes place from mid-April to late May. (Jia et al., 2023). In Fujian, the spring tea season often coincides with the rainy season. High-altitude tea regions (higher than 800 m) are frequently covered by dense fog, which significantly reduces the number of sunny days available for sun withering, limiting sunlight-withering opportunities to less than 30 % of the entire annual production cycle. (Jia et al., 2023). These factors hamper the normal sun withering process, which in turn affects the quality of the fresh leaves, impairs processing, reduces tea quality, and negatively impacts the economic benefits of tea farmers. Therefore, research into stable light sources that simulate sunlight has become essential.

Processing steps have a profound impact on the flavour of tea (Wu et al., 2022). Oolong tea is a typical semi-fermented tea, with preliminary processing steps that include plucking fresh leaves, withering, turning over, firing, rolling, drying, and final product preparation (Zheng et al., 2022). Withering is the initial step in the process that shapes the characteristic flavour of oolong tea (Qi et al., 2024). During withering, factors like light, temperature, humidity, and wind impact the balance of amino acids, caffeine, polyphenols, and sugars, which are crucial for the rich and smooth flavour of oolong tea (Li et al., 2025; Wang et al., 2022). The withering stage is also important for improving the quality of rain-soaked leaves. Traditional sun withering requires the average sunlight intensity for oolong tea withering, around 4–5 PM, to be about 6000 lx, with visible light wavelengths between 390 and 770 nm. In the traditional withering process for rain-drenched tea, the high ambient humidity and the elevated moisture content in the fresh leaves hinder the proper biochemical transformations within the leaves, resulting in inferior taste and lower-quality tea products (Tan et al., 2025). Light plays an indispensable role in tea processing (Deng et al., 2020), but sunlight withering is not always guaranteed. Currently, hot air withering is commonly used in place of sunlight withering to mitigate the negative effects of rainy weather on oolong tea processing, although the results are not optimal. Therefore, how to make rain-soaked leaves achieve the excellent quality of sunlight withering is the difficulty and bottleneck faced by oolong tea production.

LED light sources have high light emission purity and can be controlled. These sources have been widely used in areas like horticultural crop gene regulation and yield quality, showing good results (Bantis et al., 2018). Recent studies have highlighted the impact of LED light on tea quality, including the analysis of biochemical mechanisms such as shading and yellowing (Chen et al., 2021; Shen et al., 2024), as well as the improvement of tea quality under various light conditions (monochromatic and combined light) (Fu et al., 2015; Hua et al., 2024; Xie et al., 2025), particularly in oolong tea (Ni et al., 2023). Currently, the final quality of tea is often presented in finished tea. Fu et al. (2015) focused only on LED effects on physiological indices in fresh leaves and did not analyse finished tea flavour via multi-omics. Secondly, Hua et al. (2024) and Xie et al. (2025) focused only on the effect of single-wavelength LED light on tea quality, lacking high-throughput metabolic analysis of the combined LED light on tea quality after simulating sunlight. In addition, Ni et al. (2023) tested LED impacts on oolong tea flavour only under standard summer conditions and did not consider the high-humidity, rainy-season environment; Research by Tan et al. (2025) on rain-soaked leaves of oolong tea has focused primarily on temperature and humidity, overlooking “light” as a critical factor. Therefore, utilizing controllable artificial light sources to simulate sunlight withering and enhance oolong tea quality during rainy weather is a critical and emerging topic.

This study used common oolong tea varieties (“Shuixian,” “Ruixiang,” and “Chunlan”) as raw materials and applied multi-wavelength (380–780 nm) LED combination light (6000 lx intensity) with a control group for comparison experiments on the finished oolong tea. Sensory evaluation, tea soup colour differences, and electronic tongue technology were initially employed to confirm that LED light-withered oolong tea has a richer and smoother taste. Additionally, widely targeted metabolomics was employed to perform differential analysis of nonvolatile metabolites in the finished tea, identifying key taste compounds in oolong tea subjected to LED light withering and explaining the reasons behind the enhanced flavour. This research offers a comprehensive theoretical basis for controlling the light withering process of oolong tea during rainy periods and improving its overall quality.

## 2 Materials and methods

### 2.1 Preparation and extraction of tea samples

**Fig. 1:**
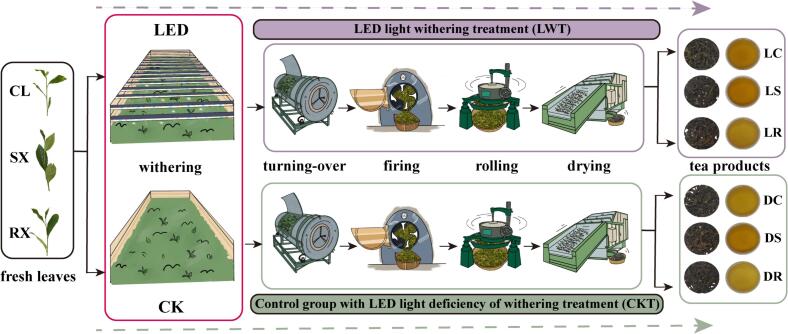
Simplified flowchart for processing oolong tea in the LWT and CKT groups. CL: Chunlan; SX: Shuixian; RX: Ruixiang; LC: ‘Chunlan’ oolong tea withered by LED light; LS: ‘Shuixian’ oolong tea withered by LED light; LR: ‘Ruixiang’ oolong tea withered by LED light; DC: ‘Chunlan’ oolong tea withered by LED light deficiency; DS: ‘Shuixian’ oolong tea withered by LED light deficiency; DR: ‘Ruixiang’ oolong tea withered by LED light deficiency; LWT group includes LC, LS and LR; CKT group includes DC, DS and DR.

In this study, the oolong tea varieties Chunlan, Shui Xian, and Rui Xiang were selected for light-withering experiments. The tea leaves of the three varieties were picked from the same location (Xiandian Village, Xingtian Town, Wuyishan City, Nanping, Fujian Province) and in accordance with the standards (healthy, disease-free, and pest-free young leaves with 3–4 open leaves), and the harvesting times were also nearly identical. As shown in Fig. 1, two withering methods were applied in this experiment: the LED light withering group (LWT) and the control group (CKT). To replicate traditional sun withering quality during rainy days, the LWT group was subjected to withering treatment using a multi-wavelength (380–780 nm) LED combination light (ZK-TB18, Sanan Sino-Science Photobiotech Co., Ltd.，Fujian，China) under a light intensity of 6000 lx (the average intensity of sunlight withering at four or five o'clock in the evening during the processing of Wuyi rock tea). The LED combination light fixtures were positioned 15 cm above the tea surface, with lamp tubes evenly spaced 10 cm apart. The CKT group was subjected to normal withering conditions without artificial LED light.

To control for as many variables as possible, all three tea varieties (LWT and CKT groups) were processed in the same workshop at the Yanba Tea Factory in Wuyishan under consistent temperature and humidity conditions (a room temperature of 26 ± 1 °C and a relative humidity of 80 ± 2 %). All the processing steps were consistent, except for the withering stage. The materials were processed into oolong tea following standard procedures: fresh leaves, withering, turning over, firing, rolling, drying, and final product preparation (Fig. 1). As a result, six tea products were obtained: ‘Chunlan’ oolong tea (LC), ‘Shuixian’ oolong tea (LS), and ‘Ruixiang’ oolong tea (LR) subjected to LED combination light withering, along with the control group products for each of the three varieties (DC, DS, and DR). Each of the six tea products included three biological replicates and was stored at −80 °C.

### 2.2 Sensorial analysis

As per the method specified in GB/T 23776–2018, we steeped 3.0 g of dry tea in boiling water for five minutes using a cylindrical cup with a tea-to-water ratio of 1:50. Based on methods from previous research (Hu et al., 2023), sensory evaluations of the tea samples were conducted by a trained panel of five senior tea assessors (three males and two females, aged 20 to 50 years). All assessors had over five years of experience in sensory analysis and had received relevant training. Then, quantitative descriptive analysis (QDA) was conducted on the LWT and CKT groups as outlined in previous studies (Peng et al., 2023; Zheng et al., 2022). Following preliminary trials and screening, the final taste evaluation indicators for oolong tea were defined as grass, bitterness, astringency, umami, sweetness, mellowness, richness, and thickness. Each indicator was rated on a scale from 0 to 9, following the method described by (Lin et al., 2024). Each sample was evaluated with three biological replicates. Its attribute intensity value was calculated as the average score from the evaluation panel. The tea samples were assessed according to GB/T 23776–2018, and ethical approval was not needed. We adhered to protocols designed to safeguard participants' rights and ensure their privacy throughout the research. Before conducting the experiment, we informed all panellists and secured their informed consent.

### 2.3 Analysis of colour differences among the tea infusions

The colorimeter (YS3060, San'enshi Technology Co., Ltd., Shenzhen, China) employs a D65 standard light source and a 10-degree observation mode for planar measurements. Based on previous research (Wu et al., 2023), the colorimeter was calibrated for black-and-white before testing. Measurements were conducted at a controlled temperature of 25 ± 1 °C in a temperature-regulated environment. All tea soups were cooled to room temperature (25 ± 1 °C) prior to measurement. Pure water was used as the reference standard, and the colour parameters of the tea infusion were measured by scanning three times, with the average value calculated. We used the Hunter colour values L*, a*, and b* to measure the colour. The value L* ranges from black (0) to white (100), indicating brightness. Meanwhile, a* shows the shift between red (+a*) and green (−a*). Similarly, b* reflects the transition from yellow (+b*) to blue (−b*).

### 2.4 Electronic tongue (*E*-tongue) analysis

The iTongue30 electronic tongue system (THINKSENSO & SENSO) was used in this study. The system has five sensors, each corresponding to one of the five basic taste factors: sourness, saltiness, umami, sweetness, and bitterness. The sensor of the electronic tongue was made of an inert precious metal, and it was unaffected by temperature and humidity. The instrument was activated to establish the database, and the sensors were immersed in the sample solution for preheating for more than 1 h. Parameter settings: One sample was selected from each group, and the “Start” or “Pretest” button was clicked to adjust the sensitivity according to the sampling signal. Following previous methods (Lin et al., 2022), 3 g of tea products were brewed for 4 min in 150 mL evaluation cups, with each sample being repeated three times to obtain the test samples. The standard reference solution was a 3 mol/L potassium chloride solution. Drift correction was performed through cross detection of the sample (Mao et al., 2024). Additionally, standard polishing techniques were employed to clean and polish the sensors before each test. After the sensors were cleaned, they were immersed in the test samples, and the experiment was initiated. After each experiment, the sensor was cleaned as required to ensure maximum stability during testing.

### 2.5 Comprehensive metabolomic assessment of nonvolatile substances in LWT and CKT groups

The samples were analyzed by MetWare Biotechnology Co., Ltd. (Wuhan, China) with minor modifications based on prior research (Wu et al., 2020). In summary, we used a freeze-dryer (Scientz-100F) to freeze-dry the samples for 63 h. Afterward, we ground them into powder with a grinder (MM 400, Retsch) operating at 30 Hz for 1.5 min. Then, 50 mg of the sample powder was weighed using an electronic balance (MS105DΜ), mixed with 1200 μL of 70 % methanol-water internal standard extraction solution, and precooled at −20 °C. The sample solution was vortexed six times, with each vortex lasting 30 s and occurring every 30 min. Subsequently, it was centrifuged at 12,000 rpm for 3 min. The supernatant was then carefully aspirated and filtered through a 0.22 μm membrane into a sample vial.

The extracts were analyzed using an UPLCESI-MS/MS system, which consisted of an UPLC (ExionLC AD; Applied Biosystems Sciex, Foster City, CA, USA) and a mass spectrometer (Applied Biosystems Sciex 4500 QTRAP), following the protocol outlined in prior research (Li et al., 2024). Specifically, the operational parameters of the ESI source were configured as follows: the source temperature was set to 500 °C, and the ion spray voltage (IS) was 5500 V for positive ion mode and − 4500 V for negative ion mode. The pressures for ion source gas I (GSI), gas II (GSII), and curtain gas (CUR) were set at 50 psi, 60 psi, and 25 psi, respectively. High collision-activated dissociation (CAD) was applied. Multiple reaction monitoring (MRM) scans were performed in the QQQ mode, with the collision gas (nitrogen) maintained at a medium level. Resolution (linear ion trap): 7070 FWHM at *m*/*z* 2122, scanning rate of 250 amu/s. The declustering potential (DP) and collision energy (CE) for each individual MRM transition were optimized. Specific MRM transitions were continuously monitored during each period based on the metabolites eluted during that time frame.

The qualitative analysis of metabolites was conducted based on retention time (RT), MS2 fragments, MS2 fragment isotope distribution, accurate m/z values, and the self-built MetWare Database (MWDB, V4.5 version, MetWare Metabolic Biotechnology Co., Ltd., Wuhan, China). The MS tolerance and MS2 tolerance were set to 20 ppm, and the RT tolerance was 0.2 min. Quantitative analysis was performed using multiple reaction monitoring (MRM) with triple quadrupole mass spectrometry. In MRM mode, the quadrupole first screened the precursor ions of the target substance. The precursor ions were then ionized in the collision chamber, resulting in the formation of multiple fragment ions. These fragment ions were filtered through a triple quadrupole to isolate the desired characteristic fragment ion. Following the acquisition of mass spectrometry data for metabolites from various samples, the chromatographic peak areas were integrated. Additionally, the peak areas for identical metabolites across different samples were normalized through peak integration, as described by Fraga et al. (2010). To assess the reproducibility and reliability of the analysis results, each sample powder was mixed into three portions to prepare quality control (QC) samples.

### 2.6 Data analysis

First, a *t-*test was performed on the electronic tongue and colour difference data using SPSS 26.0 software to assess statistical significance and identify differences between treatment groups. Second, multiple statistical methods were applied to the metabolite data. Principal component analysis (PCA) and orthogonal partial least squares discriminant analysis (OPLS-DA) were conducted using R version 3.5.1 software with the default settings. Hierarchical cluster analysis (HCA) was performed using the TBtools software. Differentially abundant metabolites were identified by applying both OPLS-DA and fold change (FC) filtering methods. Metabolites with a variable importance in projection (VIP) ≥ 1 and an FC ≥ 1.6 were considered significantly upregulated, while those with a variable importance in projection (VIP) ≥ 1 and an FC ≤ 0.62 were deemed significantly downregulated. Finally, calculations and figure creation were carried out using Excel 2016, GraphPad Prism 9.0, and Adobe Illustrator 2023 software.

## 3 Results and discussion

### 3.1 Comparison of sensory and phenotypic characteristics between the LWT and CKT groups

#### 3.1.1 Sensory quality analysis of LED light-withered oolong tea

We conducted sensory evaluations on the LWT and CKT groups (Table 1) to analyse the impact of LED light withering treatment on the flavour of three representative oolong tea products. The colour of the liquor in the LWT group was slightly darker than that in the CKT group, suggesting that the difference in colour may be attributed to variations in nonvolatile compounds. All the samples displayed the typical floral and fruity aromas of oolong tea, but the LWT group presented a more distinct fragrance overall. Notably, regarding taste, the CKT group had a slightly astringent flavour, whereas the LWT group was richer and featured a floral note, suggesting that LED light withering treatment enhanced the development of a mellow, thick flavour in oolong tea.

**Table 1:** Sensory evaluation results of the LWT and CKT groups.

Treatment	Sample	Liquor colour		Aroma		Taste	
		Comment	Score	Comment	Score	Comment	Score
CKT	DC	Light orange-yellow, bright	86.82 ± 0.66 ^e^	Pure, flower aroma	87.92 ± 0.41 ^e^	Thick but with grass and astringency	86.96 ± 0.59 ^e^
LWT	LC	Orange-yellow, bright	88.04 ± 0.32 ^d^	High and pure, flower aroma	90.02 ± 0.38 ^d^	Thick and mellow	91.16 ± 0.27 ^c^
CKT	DS	Light orange-red, bright	88.88 ± 0.52 ^c^	Clean, flower aroma	89.94 ± 0.51 ^d^	Mellow but with grass and astringency	89.82 ± 0.40 ^d^
LWT	LS	Orange-red, bright	90.00 ± 0.43 ^b^	High and clean flower aroma	93.08 ± 0.67 ^b^	Thick and mellow, soup with floral aroma	92.52 ± 0.40 ^b^
CKT	DR	Light orange-yellow	89.98 ± 0.59 ^b^	Rich aroma, with peach-like fragrance	91.98 ± 0.50 ^c^	Mellow and fresh but with astringency	90.90 ± 0.75 ^c^
LWT	LR	Light orange-yellow, bright	91.16 ± 0.74 ^a^	Rich aroma, with fruity fragrance	94.06 ± 0.71 ^a^	Mellow and fresh, soup with floral aroma	93.48 ± 0.87 ^a^

Note: The scores for the data are shown as averages with standard deviations (*n* = 5). Each attribute is assessed on a scale of 100. In the indicators of liquor colour, aroma, and taste, the different superscript lowercase letters represented significant difference (*p* < 0.05) among oolong tea samples.

**Fig. 2:**
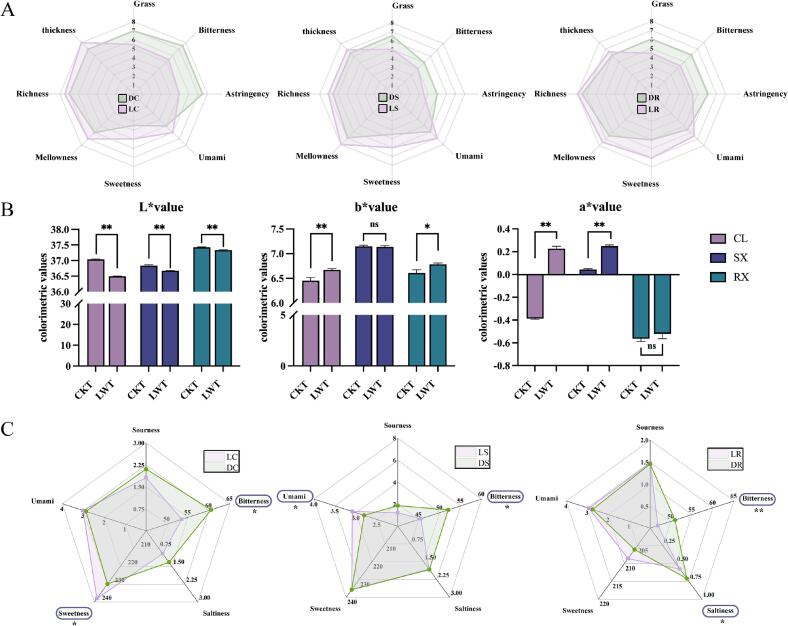
Profiles of taste sensory visualization of oolong tea samples from the LWT and CKT groups. * represents a significant difference (*p* < 0.05); ** represents a highly significant difference (*p* < 0.01); ns: no significant difference. (A) Sensory quantitative descriptive analysis (QDA) results for the LWT and CKT groups. (B) Colour difference analysis results for the LWT and CKT groups. CL: Chunlan; SX: Shuixian; RX: Ruixiang; CKT: Control group without LED light during the withering treatment; LWT: LED light withering treatment. (C) Electronic tongue analysis results of the LWT and CKT groups.

The results revealed significant differences in taste between the LWT and CKT groups. We conducted further taste analysis using QDA, evaluating eight characteristic taste profiles. As shown in Fig. 2A, the green, bitterness, and astringency indices of the LWT group were significantly lower than those of the CKT group. Specifically, the taste concentration of the LC was significantly thicker, and the mellowness of the LS was notably enhanced. Notably, LR presented a “watery fragrance,” and the richness of the tea liquor was significantly increased. In conclusion, the overall quality of the LWT group was greater than that of the CKT group, with particularly notable differences in liquor colour and taste.

#### 3.1.2 Analysis of the influence of LED light withering on colour differences in oolong tea soup

To further investigate the macroscopic effects of LED light withering on oolong tea, we used the colour indices L*, a*, and b* to analyse the colour differences between the LWT and CKT groups. As shown in Fig. 2B, the L* values of the LWT group were significantly lower than those of the CKT group. The b* values of the LWT group increased, with highly significant and significant differences observed in CL and RX, respectively, indicating a deepening of the yellow colour in the LWT tea liquor. Similarly, the a* values of the LWT group increased compared with those of the CKT group, with highly significant differences observed in CL and SX. Specifically, after treatment, the colour of CL shifted from green to red, the red colour of SX deepened, and the green colour of RX lightened, suggesting that the red colour of the LWT group tea liquor deepened, whereas the green colour weakened. Overall, the colour of the LWT group darkened, showing an orange-yellow to orange-red hue, which was different from the CKT group and matched the results of the sensory evaluation. Under abiotic stress, secondary metabolites may trigger stress resistance responses or act as signalling molecules (Wu et al., 2022). This effect may be due to the transformation of secondary metabolites under LED light treatment, leading to different accumulation patterns of pigments like theaflavins and thearubigins.

#### 3.1.3 Electronic tongue analysis of LED light-withered oolong tea

To reduce the influence of subjective factors on taste results in sensory evaluation, we did electronic tongue measurements on the LWT and CKT groups to compare the flavour effects of LED light withering on oolong tea. The results showed that the bitterness value of the LWT group was lower than that of the CKT group, with clear changes in the taste profile (Fig. 2C), which matched the results of the QDA. Specifically, LC had a higher sweetness value than DC (*p* < 0.05), and LS had a higher umami value than DS (p < 0.05). Compared with DR, LR had a lower bitterness value and a higher saltiness value (*p* < 0.01 and *p* < 0.05, respectively). Although no significant differences were observed in sweetness or umami values, both were slightly greater in LR than in DR. Stress-induced starch degradation increases progressively during tea processing, followed by the synthesis of amino acids or secondary metabolites to counteract adverse environmental conditions (Baba et al., 2017). This effect may be attributed to the transformation of secondary metabolites in oolong tea under LED light withering, resulting in distinct accumulations of amino acids, flavonoids, and carbohydrates.

In conclusion, we speculated that LED light withering primarily enhances the transformation of bitter compounds in oolong tea while promoting the development of mellowness through synergistic changes in umami and sweetness compounds in varying proportions. Consequently, we conducted metabolomics research to investigate the underlying mechanism involved.

### 3.2 Identification of key nonvolatile metabolites in LED light-withered oolong tea

#### 3.2.1 Dynamic changes in nonvolatile metabolites in the LWT and CKT groups

**Supplementary Fig. 1:**
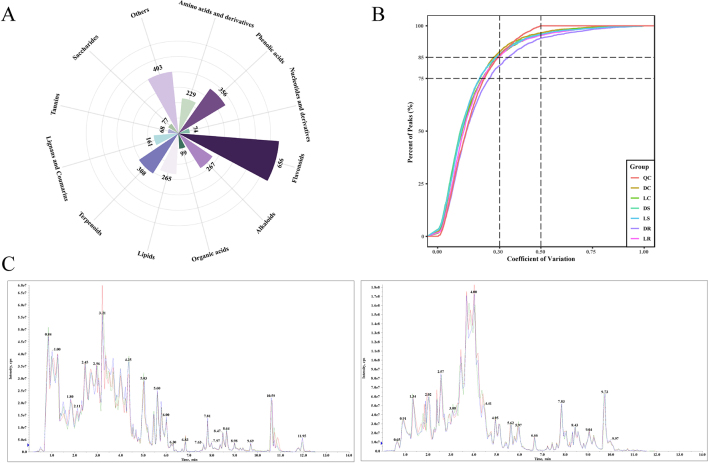

Nonvolatile metabolites play crucial roles in determining the taste of tea. Therefore, we performed widely targeted metabolomic analysis on the LWT and CKT groups. We detected a total of 2963 metabolites, including 656 flavonoids, 229 amino acids and derivatives, 356 phenolic acids, 267 alkaloids, 265 lipids, 99 organic acids, 74 nucleotides and derivatives, 161 lignans and coumarins, 68 tannins, 77 saccharides, 308 terpenoids, and 403 other compounds **(Fig. S1A)**. Flavonoids and phenolic acids were the predominant metabolites in both the LWT and CKT groups, contributing to the overall taste quality. Additionally, the total ion chromatograms (TICs) of the QC samples showed a high degree of overlap, with over 85 % of the compounds exhibiting a coefficient of variation (CV) < 0.3 **(Fig. S1B—C)**, demonstrating the high repeatability and reliability of the data.

**Fig. 3:**
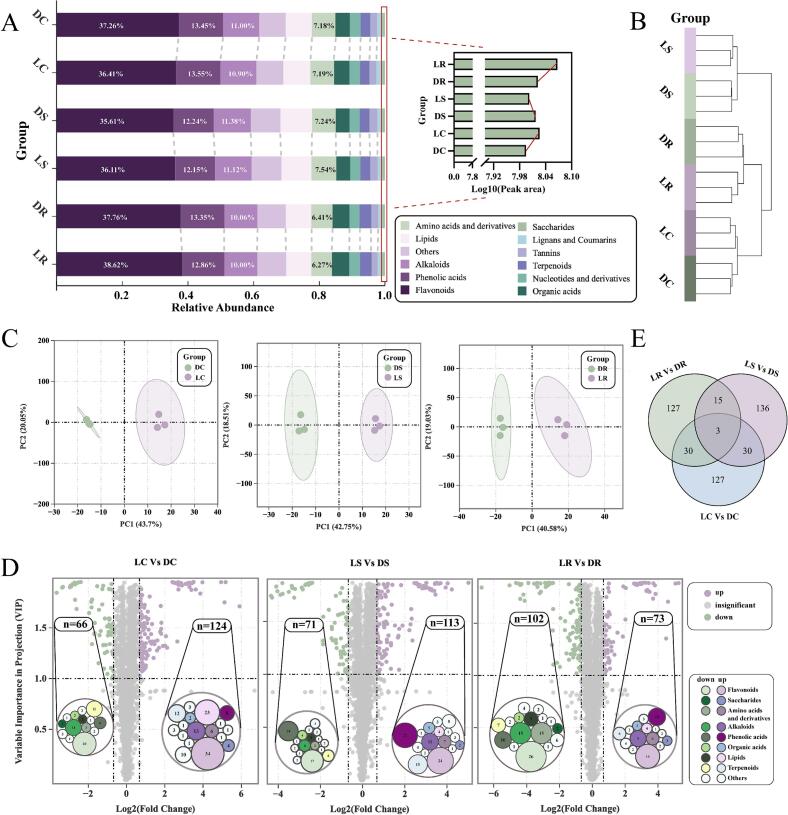
Profiles of nonvolatile metabolites from the LWT and CKT groups based on metabolomics datasets. (A) A stacked plot depicting the distribution of all nonvolatile categories across different samples. (B) A heatmap displaying clustering between different samples. (C) PCA score plot among the three groups based on the metabolomics dataset. (D) A differential volcano bubble chart depicting the number of significantly differentially abundant metabolites in various classes with the criteria of a fold change ≥1.6 or ≤ 0.62, VIP ≥ 1.0. up: upregulated metabolites; down: downregulated metabolites; insignificant: nonsignificant metabolites. The numbers in the circles of each bubble chart represent the number of characteristic metabolites of different categories. (E) Venn diagram screening for common and unique differential substances across the three sample groups.

We next compared the total peak areas of the 12 compound categories between the LWT and CKT groups **(**Fig. 3A**)**. Flavonoids, phenolic acids, and amino acids made up the largest proportion and exhibited fluctuations in both the LWT and CKT groups. These compounds are important in determining the flavour of tea (Zhang et al., 2020). The findings suggest that LED light withering may affect the synthesis and breakdown of key taste compounds in oolong tea. Interestingly, the total peak area of alkaloids in the LWT group was lower than that in the CKT group, whereas the total peak areas of sugars in LR and LC were greater than those in DR and DC. This could explain the increase in the sweetness index and the decrease in the bitterness index observed on the electronic tongue. Notably, the total peak area of sugars in LS was lower than in DS, but sensory evaluation showed that LS had a significantly higher sweetness than DS. Previous studies have shown that the sweetness index of tea results from the synergistic interaction of various substances (Troszyńska et al., 2010), which partly explains the phenomenon observed in this study.

The hierarchical clustering analysis grouped the samples into three distinct clusters **(**Fig. 3B**)**. Specifically, LS was grouped with DS, LR with DR, and LC with DC, while the three biological replicates of each sample formed tight clusters. These findings indicated that the LWT and CKT groups presented consistent clustering trends, and the data exhibited good reproducibility. We also performed pairwise PCA to visualize the differences between the LWT and CKT groups **(**Fig. 3C**)**. The results revealed that the first two principal components (PC1 and PC2) explained approximately 60 % of the variance, accounting for most of the variation. Notably, the LWT and CKT groups clearly separated along PC1 (accounting for approximately 40 %), with the replicates of each sample tightly clustered. These findings suggested that the nonvolatile components in oolong tea significantly changed after LED light withering, and the data were reliable, which was consistent with the results of hierarchical clustering.

#### 3.2.2 Dynamic changes in characteristic taste components in the LWT and CKT groups

To identify the significant differences in nonvolatile compounds in oolong tea after LED light withering, based on OPLS-DA, we used FC and VIP values to jointly select differentially expressed metabolites (DEMs) between the LWT and CKT groups **(**Fig. 3D**)**. A total of 465 DEMs were selected, with similar numbers across the three comparison groups. These findings suggest that the overall impact of LED light withering on different oolong tea varieties was stable. The differences in substance categories between the upregulated and downregulated trends may explain the slight variations in the taste indices observed on the electronic tongue **(**Fig. 2C**)**. The number of up-regulated metabolites in each substance category was mostly higher than that of down regulated metabolites in the difference comparison groups (LC vs DS and LC vs DS), suggesting that LED light withering increased the richness of taste compounds. This could explain the increased thickness and mellow flavours observed in the sensory evaluation. Notably, upregulated and downregulated flavonoids in the LWT and CKT groups accounted for the largest proportion of all categories, which is consistent with previous reports (Wang et al., 2024) and indicates that flavonoids undergo significant changes during the withering stage.

To gain a clearer understanding of how LED light withering influences the nonvolatile compounds in oolong tea, we used a Venn diagram to illustrate the relationships among the three comparison groups. As shown in Fig. 3E, the unique and shared categories of the differentially expressed metabolites were consistent across the three comparison groups, with flavonoids, phenolic acids, alkaloids, and amino acids being the predominant classes. Therefore, we hypothesize that LED light withering primarily affects the flavour of oolong tea by altering the contents of sweet, bitter, and astringent compounds.

### 3.3 Construction of a taste metabolism network for LED light-withered oolong tea via joint pathway analysis

**Fig. 4:**
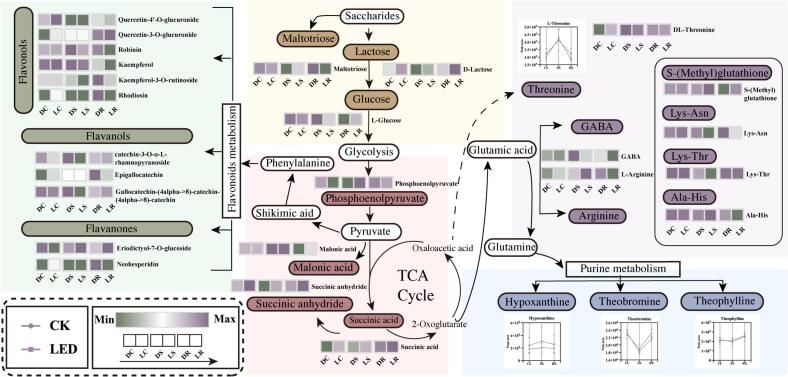
A predictive network diagram of metabolic pathways related to flavour formation in LED light-withered oolong tea was constructed by drawing line charts and heatmaps among the three sets of comparisons. The heatmap displays characteristic substances (fold change ≥1.6 or ≤ 0.62, VIP values ≥1.0). The line chart presents insignificant but important taste substances.

**Supplementary Fig. 2:**
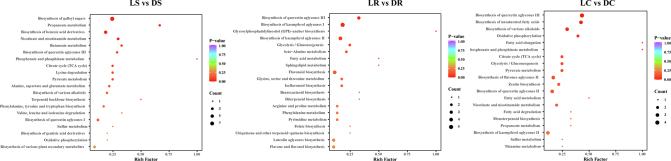

Multiple pathways are typically involved in generating nonvolatile metabolites. The DEMs underwent KEGG enrichment analysis to pinpoint significant metabolic pathways. The results revealed that metabolic pathways were enriched mainly in glycolysis/gluconeogenesis (ko00010), biosynthesis of various alkaloids (ko00996), propanoate metabolism (ko00640), flavonoid biosynthesis (ko00941), and amino acid metabolism {lysine degradation (ko00310), alanine, aspartate, and glutamate metabolism (ko00250), glycine, serine, and threonine metabolism (ko00260), etc.} **(Fig. S2)**. These findings suggested that the different varieties of oolong tea presented high metabolic similarity after being subjected to LED light withering. Therefore, this study integrated substance content, thresholds, and flavour characteristics to illustrate the changes in important and key nonvolatile metabolites in LED light-withered oolong tea through metabolic pathways **(**Fig. 4**)**. Based on enrichment analysis and prior studies (Guo et al., 2023; Yu & Yang, 2020; Zhang et al., 2020), we examined the major taste compounds, including amino acids, flavonoids, soluble sugars, alkaloids, and organic acids.

Soluble sugars, as initial products of photosynthesis, significantly contribute to the sweetness of tea, and studies on rainwater oolong tea have also been conducted (Tan et al., 2025). Monosaccharides and disaccharides are the main soluble sugars in tea. As shown in Fig. 4, d-lactose, the major disaccharide in tea, increased in all LWT groups. LC was significantly greater than DC by a factor of 1.72. l-glucose, the main monosaccharide in tea, was 1.71 times higher in LR than in DR. Maltotriose is a short-chain glucose oligosaccharide, and recent studies have shown that it has a mild sweetness (Pullicin et al., 2019). Its concentration in LS was significantly (7.33 times greater) than that in DS. Additionally, the contents of some soluble sugars in the LWT group decreased, although no significant differences were detected. This could be attributed to sucrose and its hydrolysis byproducts functioning as signalling molecules. These compounds are detected and transmitted via the glycolysis pathway, leading to the accumulation of stress-related molecules (Baba et al., 2017). In summary, the high content of major soluble sugars may explain why the sweetness in the LWT group was greater than that in the CKT group, which is consistent with the electronic tongue results.

Photosynthetic products are oxidized and metabolized through respiration, resulting in the formation of various keto acids and other organic acids. Phosphoenolpyruvate (PEP) plays essential roles in organisms, including in glycolysis, photosynthesis, and cellular protection, among other functions (Xue et al., 2024). As shown in Fig. 4, the phosphoenolpyruvate (PEP) content changed after LED light withering. Its content in LR was 9.97 times lower than in DC, and in LS was 16.34 times higher than in DS. These findings suggest that LED light withering may affect the taste of oolong tea by influencing different taste-related metabolic pathways. Malonic acid, which comes from pyruvate, helps balance bitterness. Its content in LR was up to 11.19 times greater than in DR. Notably, both succinic acid and succinic anhydride increased in the LWT group, reaching 1.73 to 5.90 times higher than the levels in the CKT group. Studies have shown that succinic acid and succinic anhydride can improve taste richness and smoothness (Shan et al., 2024). Succinic acid accumulation may be caused by Light-induced TCA cycle bypass via GABA shunt (Eprintsev et al., 2024). This may also be one of the reasons for the decrease in GABA content in the results. In conclusion, LED light withering may increase metabolic activity, and the accumulation of organic acids that mask bitterness could be one potential reason for the greater richness in the LWT group than in the CKT group.

Flavonoid compounds from phenylalanine metabolism are important contributors to bitterness in tea. Specifically, flavanols and flavonols are key taste components that contribute to mild astringency and have low threshold values (Fang et al., 2019). In the LWT group, most flavanols and flavonols underwent hydrolysis due to heat and enzymatic action (Fig. 4), which partially reduced the bitterness of the oolong tea. Notably, catechin-3-O-α-L-rhamnopyranoside and kaempferol-3-O-robinoside-7-O-rhamnoside (robinin) were significantly downregulated and were common differential compounds between the LWT and CKT groups. Specifically, the content of catechin-3-O-α-L-rhamnopyranoside was 1.92 times lower in LC than in DC and 20.23 times lower in LS than in DS. The content of robinin was 1.98 times lower in LC than in DC and 19.53 times lower in LR than in DR. The LED light may alter electron transport chain activity in chloroplasts, leading to transient NADPH/H₂O₂ fluctuations (Ugalde et al., 2021). Elevated H₂O₂ could activate aldo-keto reductases (e.g., AKR1A1), as reported in Zeng et al. (2017). This enzyme family is known to catalyse the reduction of carbonyl groups in flavonoids. We also acknowledged that further enzyme activity assays were needed to confirm this. Changes in key flavonoid compounds after LED light-withering followed common patterns, with the downregulation of these compounds contributing to the reduction of bitterness in LED light-withered oolong tea.

Owing to the threshold and flavour characteristics of flavonoids (Guo et al., 2023), kaempferol, kaempferol-3-O-rutinoside, epigallocatechin, rhodiosin, and hesperetin-7-O-neohesperidoside (neohesperidin) presented low thresholds and bitterness. These compounds tended to decrease in the LWT group, particularly in the LR group **(**Fig. 4**)**. Additionally, the content of eriodictyol-7-O-glucoside in the LC group was 3.55 times lower than that in the DC group. In the LS group, the amount of gallocatechin-(4alpha- > 8)-catechin-(4alpha- > 8)-catechin was 13.97 times lower than that in the DS group. We hypothesized that the LED light withering process affects the flavonoids in various oolong tea varieties differently but consistently reduces the levels of key bitterness compounds. Velvet-like astringency is considered a positive sensation, often described as “velvety” or “silky” (Guo et al., 2023), contributing to the tea's richness. Interestingly, the quercetin-4’-O-glucuronide and quercetin-3-O-glucuronide, both of which are linked to velvet-like astringency, was increased in the LWT group, and particularly notable in LR. These findings suggest that LED light withering may promote the accumulation of the specific flavonoid glycosides that are responsible for velvet-like astringency. In conclusion, LED light withering promoted the breakdown of most flavonoids while increasing the specific flavonoid aglycones responsible for velvet-like astringency. This may explain the observed reduction in bitterness and the increased richness and yellow hue of the LWT group.

Theobromine and caffeine are key bitter compounds in tea derived from hypoxanthine in the tea leaves. These compounds interconvert in purine alkaloid metabolism and interact with various substances, influencing the flavour of tea (Song et al., 2025). As shown in Fig. 4, there were no significant differences in the contents of these three compounds between the LWT and CKT groups (none met the criteria of VIP ≥ 1, FC ≥ 1.6 or ≤ 0.62), which was consistent with previous studies indicating that the levels of alkaloids, such as caffeine, remain relatively stable during tea processing (Chen et al., 2020). Interestingly, in the LWT group, hypoxanthine levels were higher than those in the CKT group, whereas theobromine and caffeine levels were slightly lower in the LWT group overall than in the CKT group. Therefore, we hypothesized that most alkaloids formed during the LED light withering process may contribute to the formation of tea flavour through interactions with other substances, warranting further investigation.

Amino acids, their concentration, composition, and transformation products significantly affect the quality of tea. *S*-methylglutathione primarily contributes to antioxidant and stress resistance functions (Kalinina & Novichkova, 2021; Short et al., 2016). As shown in Fig. 4, the *S*-methylglutathione levels in the LWT group were greater than those in the CKT group, especially in LC, where they were 13.17 times greater than those in DC. Thus, we hypothesize that LED light withering may regulate tea plant metabolism through various signalling pathways, indirectly enhancing both the quality and yield of tea. Certain amino acids play a crucial role in determining the sweetness, bitterness, and freshness of tea, making them essential components for assessing tea quality. Interestingly, a combination of the taste properties and threshold of amino acids (Scharbert & Hofmann, 2005; Yu & Yang, 2020) revealed that substances such as L-threonine, L-arginine, and γ-aminobutyric acid presented lower threshold and different contents, which are key differential substances between the LWT group and the CKT group. As shown in Fig. 4, the concentrations of sweet amino acids such as L-threonine and DL-threonine increased in the LWT group, with the most significant increase observed in LC. This may explain the greater sweetness observed in the LWT group than in the CKT group. Notably, bitter amino acids such as L-arginine and γ-aminobutyric acid were key differentiators between the LWT and CKT groups, with their concentrations generally decreasing in the LWT group (Fig. 4). Many oligopeptides exhibit greater bitterness than individual amino acids do, and many amino acids and oligopeptides have synergistic effects on bitterness (Yan et al., 2025). As shown in Fig. 4, the levels of most oligopeptides were significantly lower in the LWT group than in the CKT group. In conclusion, the reduction in key bitter amino acids and oligopeptides may underlie the decreased bitterness observed in the LWT group compared with the CKT group.

## 4 Conclusion

This study combined sensory omics and widely targeted metabolomics to analyse the taste characteristics and nonvolatile compound changes in the LWT and CKT groups. Key metabolites were identified to construct a visualized taste pathway network. Sensory evaluation, colour differences, and electronic tongue results indicated that LED light withering increased the richness of the flavour of oolong tea and deepened the liquor colour to produce orange-yellow and orange-red shades. Widely targeted metabolomics results showed that the effect of LED light withering on the taste of oolong tea was mainly mediated via promoting the transformation of bitter substances such as flavonoids (kaempferol-3-O-rutinoside, kaempferol, and robinin), alkaloids, bitter amino acids (L-arginine and γ-aminobutyric acid), and oligopeptides, supplemented by different proportions of umami and sweet substances, thereby promoting the formation of a rich and mellow taste. This study establishes a theoretical foundation for controlling light-withering and improving the quality of oolong tea under rainy conditions, while also suggesting potential directions for further process optimization. Despite these achievements, there has been a gap in the targeted quantification of essential taste compounds, as well as in the investigation of how the ratios of these substances influence taste perception. This limitation prevents a deeper understanding of the key taste compounds in oolong tea subjected to LED light withering. Future studies could explore taste recombination, enzyme activity assays and perform molecular docking experiments. However, our study systematically explored the improvement of oolong tea taste by using multi-wavelengthLED combination light from the perspectives of sensory omics and metabolomics. This study developed a comprehensive metabolic network from key flavour compounds, including soluble sugars, organic acids, flavonoids, and amino acids, aiming to elucidate the relationship between the changes in compounds and the quality enhancement of rain-soaked oolong tea after controllable light-withering.

The following are the supplementary data related to this article.

## CRediT authorship contribution statement

**Qiuming Li:** Writing – original draft, Formal analysis, Data curation, Conceptualization. **Jihang He:** Writing – review & editing. **Xinru Yu:** Writing – review & editing. **Yongpeng Wu:** Resources, Project administration. **Kang Li:** Resources, Project administration. **Zongjie Wu:** Writing – review & editing. **Yucheng Zheng:** Writing – review & editing, Methodology. **Yun Sun:** Writing – review & editing, Resources, Project administration, Methodology. **Hongzheng Lin:** Writing – review & editing, Project administration, Methodology.

## Declaration of competing interest

The authors confirm that they have no known financial interests or personal relationships that could be perceived as influencing the research presented in this paper.

## Data Availability

Data will be made available on request.
